# Spread of Health Risk Factors among Adolescents from Plovdiv, Bulgaria

**DOI:** 10.18502/ijph.v49i8.3904

**Published:** 2020-08

**Authors:** Elena MERDZHANOVA, Gergana PETROVA, Maria VAKRILOVA-BECHEVA

**Affiliations:** Department of Nursing, Faculty of Public Health, Medical University of Plovdiv, Plovdiv, Bulgaria

## Dear Editor-in-Chief

Adolescent years have become a starting point for the formation of health behaviors that have lifetime consequences (1, 2). Adolescent age (adolescent, teenage) is a transition period from child to adulthood (3). ‘Healthy lifestyle habits during adolescence can prevent many of the diseases and disabilities in adulthood and later’ (4). A healthy lifestyle is a system of activities consciously aimed at strengthening, preserving and maintaining the health of both the individual and society (5).

This study aimed to analyze the distribution of health risk factors - smoking and alcohol among adolescents from Plovdiv, Bulgaria. The study was conducted at 17 schools on the territory of Plovdiv in two consecutive academic years: 2015/16 and 2016/17. Regular pupils are aged 11–14 yr old. Overall, 302 students were enrolled in the general study. The type of sociological survey is a direct group survey. Based on the purpose of the study, as well as on the volume and type of the data, the statistical methods used were: descriptive statistics and alternative analysis. The χ^2^ and Fisher’s exact test criteria were applied.

The scientific work has been presented to The Scientific Ethics Committee on Sep 21, 2017, which gave its opinion with an order of The Rector of Medical University - Plovdiv № P-2550 / 12.10.2017.

Nineteen (6.9%) of all reported to have started smoking with a slightly higher prevalence for boys 11 (7.7%) and 8 (6.1%) for girls, with gender of no significance. Immediately, there arose the question about the level of adolescents’ awareness of the health-related consequences of smoking, with 205 (74.5%) claiming to be informed, of whom 106 (73.6%) boys and 99 (75.6%) girls. The remaining 50 (25.4%) – 15 (10.4%) boys and 14 (10.7%) girls declared not to have been informed or having been only partially informed - 23 (16%) boys and 18 (13.7%) girls. Despite differences in the relative proportions of responses, gender is not relevant to adolescent awareness. Protection from tobacco smoke reported more boys - 40 (27.8%) than girls 17 (13%), the former were less than half of all boys (x^2^ = 9.537; df = 2; *P* = 0.0008).

Another risk factor with its negative effect - alcohol, was also the object of the study and the issues related to it presented the following results. Of all respondents, 19 (6.9%) used alcohol with boys - 14 (9.7%) outnumbering girls - 5 (3.8%). When comparing the two genders, it is obvious that the majority of boys using alcohol 7 (53.8%) made it relatively rare (less than once - twice a month) whereas girls in the same group were only 2 (40%). There was a significant difference in the daily use of alcohol, which occurs only with girls and in alcohol consumption of 2–3 times a week and 2–3 times a month, which was only declared by boys. Gender had statistical significance in alcohol consumption as more girls consume alcohol than boys do (x^2^ = 3.719; df = 1; *P*= 0.05), but no significant prevalence was demonstrated.

Girls are less controlled by their parents regarding health risk factors, they use more harmful substances (cigarettes and alcohol) compare to boys. The main sources of information for the adolescents are the family and the school.
